# Functional relationship between matrix metalloproteinase-11 and matrix metalloproteinase-14

**DOI:** 10.1002/cam4.290

**Published:** 2014-08-01

**Authors:** Emilie Buache, Robert Thai, Corinne Wendling, Fabien Alpy, Adeline Page, Marie-Pierre Chenard, Vincent Dive, Marc Ruff, Annick Dejaegere, Catherine Tomasetto, Marie-Christine Rio

**Affiliations:** 1 Institut de Génétique et de Biologie Moléculaire et Cellulaire, Department of Functional Genomics and Cancer, CNRS UMR 7104, INSERM U964, Université de StrasbourgIllkirch, France; 2 Commissariat à l'Energie Atomique, Service d'Ingénierie Moléculaire des Protéines (SIMOPRO)Gif/Yvette, 91191, Cedex, France; 3 Institut de Génétique et de Biologie Moléculaire et Cellulaire, Plateforme de protéomiqueIllkirch, France; 4 Service d'Anatomie Pathologique Générale, Centre Hospitalier Universitaire de HautepierreStrasbourg, France; 5 Institut de Génétique et de Biologie Moléculaire et Cellulaire, Department of Structural Biology and Genomic, CNRS UMR 7104, INSERM U964, Université de StrasbourgIllkirch, France; 6 Equipe Labellisée Ligue contre le CancerFrance

**Keywords:** Cell microenvironment, epithelial-stromal cell interaction, MMP inactivation, tissue remodeling

## Abstract

MMP-11 is a key factor in physiopathological tissue remodeling. As an active form is secreted, its activity must be tightly regulated to avoid detrimental effects. Although TIMP-1 and TIMP-2 reversibly inhibit MMP-11, another more drastic scenario, presumably via hydrolysis, could be hypothesized. In this context, we have investigated the possible implication of MMP-14, since it exhibits a spatiotemporal localization similar to MMP-11. Using native HFL1-produced MMP-11 and HT-1080-produced MMP-14 as well as recombinant proteins, we show that MMP-11 is a MMP-14 substrate. MMP-14 cleaves MMP-11 catalytic domain at the PGG(P1)-I(P1′)LA and V/IQH(P1)-L(P1′)YG scissile bonds, two new cleavage sites. Interestingly, a functional test showed a dramatical reduction in MMP-11 enzymatic activity when incubated with active MMP-14, whereas inactive point-mutated MMP-14 had no effect. This function is conserved between human and mouse. Thus, in addition to the canonical reversible TIMP-dependent inhibitory system, irreversible MMP proteolytic inactivation might occur by cleavage of the catalytic domain in a MMP-dependent manner. Since MMP-14 is produced by HT-1080 cancer cells, whereas MMP-11 is secreted by HFL1 stromal cells, our findings support the emerging importance of tumor-stroma interaction/cross-talk. Moreover, they highlight a Janus-faced MMP-14 function in the MMP cascade, favoring activation of several pro-MMPs, but limiting MMP-11 activity. Finally, both MMPs are active at the cell periphery. Since MMP-14 is present at the cell membrane, whereas MMP-11 is soluble into the cellular microenvironment, this MMP-14 function might represent one critical regulatory mechanism to control the extent of pericellular MMP-11 bioavailability and protect cells from excessive/inappropriate MMP-11 function.

## Introduction

MMP-11 (also named stromelysin-3) belongs to the MMP extracellular enzyme family 1. MMP-11 plays a role in embryonic implantation, organ ontogenesis, tissue involution, repair processes 2,3, and numerous diseases like atherosclerosis, rheumatoid arthritis, and cancers 4,5. In all cases, MMP-11 is produced by cells of mesenchymal origin mainly fibroblast-like cells and adipocytes 6 (reviewed in 7,8). MMP-11 normally acts in tissue remodeling processes which occur at epithelial/connective interfaces to regulate epithelium homeostasis 9. In carcinomas, high MMP-11 levels in the primary tumors are correlated with bad prognoses and pejorative patient outcomes 5. Using mouse tumorigenesis models, stromal MMP-11 was found to favor cancer cell survival and implantation during the early invasive steps of adjacent connective tissues 10. However, despite numerous evidence of crucial physiological and pathological functions, how MMP-11 works at the cellular and molecular level remains largely unknown. While MMPs cleave numerous substrates (e.g., extra cellular matrix (ECM) components, pro-MMPs) 11–13, only a few substrates (e.g., IGF-BP1, laminin receptor, the native alpha 3 chain of collagen VI) were shown for MMP-11 14.

MMP-11 exhibits a sharply defined spatial and temporal expression pattern, suggesting a local and rapid function 9. MMP-11 possesses a cleavage site for intracellular furin-type convertases, between its prodomain and catalytic domain. Thus, an active form of MMP-11 is secreted, while several other MMPs need to be extracellularly processed to be functional 15,16. Therefore, MMP-11 enzymatic activity should be drastically downregulated once the enzyme is no longer needed. Indeed, its inactivation might represent a critical step in the regulation of its function. It is generally accepted that MMPs are negatively regulated by a family of four endogenous proteins called TIMP-1 to TIMP-4 17,18. Indeed, TIMP-1 and TIMP-2 inhibit MMP-11 function. However, TIMPs are not specific and TIMP-mediated inhibition is reversible. A more permanent scenario may exist for tightly downregulating MMP-11 bioactivity. Interestingly, an MMP-dependent MMP-11 processing has been reported, but the nature of the MMP involved remains unknown 19. MMP-14 might be a good candidate. Similar to MMP-11, MMP-14 plays an essential role during normal tissue remodeling processes as well as during benign and malignant diseases 20–22.

In the present study, we investigated the possible functional relationship between MMP-11 and MMP-14. We demonstrate that MMP-11 is a MMP-14 substrate and identify two cleavage sites on MMP-11. Interestingly, cleavage leads to the loss of MMP-11 enzymatic activity, highlighting a way to irreversibly inactivate MMP-11 and a novel negative regulator function for MMP-14.

## Materials and Methods

### Breast cancer histology and immunohistochemistry

Paraffin-embedded sections of human breast invasive tumors were analyzed using hematoxylin and eosin staining. Immunohistochemistry was performed using the 5ST4A9 and 3H3 mouse monoclonal antibodies (1:2000; IGBMC) directed against the human MMP-11 6 and MMP-14 23 respectively, and the kit LSAB-DAB (Ventana, Tucson, AZ).

### Expression and purification of recombinant MMPs

Production and characteristics of full-length human (FL-MMP-11, Phe98-Leu488) and mouse (Phe102-Arg492) MMP-11, catalytic domain of human (Cat-MMP-11, Phe98-Gly272) and mouse (Phe102-Ser276) MMP-11, and of human MMP-14 (Cat-MMP-14, Tyr111-Arg298) were reported 24,25. The mutant recombinant MMPs was constructed in the pET3b vector (QuickChange Site-Directed Mutagenesis Kit; Stratagene, Les ulis, France) 26,27. After transformation in the nonexpressing *Escherichia coli* strain XL1-Blue and selection, the plasmids were transformed into the expressing *E. coli* strain BL21(DES)pLysS. All constructs were sequenced. Protein was recovered, refolded, and purified from inclusion bodies 27. The primers used were the following: Cat-MMP-14 m (Glu240Ala), sense 5′TGGCTGTGCATGCGCTGGGCCATGCCCTGGGGCT3′ and antisense 5′AGCCCCAGGGCATGGCCCAGCGCATGCACAGCCA3′; FL-MMP-11AV (GI to AV), GTTTGACGGGCCTGGGGCGGTGCTGGCCCATGGCTTCT sense and AGAAGCCATGGGCCAGCACCGCCCCAGGCCCGTCAAAC antisense.

### MMP enzymatic activity

The a1-PI quantitative indirect colorimetric assay was used 27. Briefly, MMPs (100 ng to 1 *μ*g) were incubated with a1-PI before addition of a-chymotrypsin (a-CT). Then, a synthetic substrate for a-CT (N-succinyl-Ala-Ala-Pro-Phe-p-nitroanilide) carrying a chromogenic moiety, was added and the optical density at 405 nm was determined (Beckman DU640 spectrophotometer, Fullerton, CA). As reported, mouse FL-MMP-11 was more efficient than human FL-MMP-11 (about 10-fold), and the MMP-11 catalytic domain more than FL-MMP-11 24,26.

We also checked the ability of wild type or point-mutated recombinant cat-MMP-14 to activate endogenous MMP-2 produced by U87MG cell line (American Type Culture Collection), using zymography. Cell culture medium incubated with either cat-MMP-14 or cat-MMP-14 m (4 h, 37°C) where loaded onto a 10% polyacrylamide gel containing 0.1% gelatin (Invitrogen Corp., Carlsbad, CA) and separated by sodium dodecyl sulphate polyacrylamide gel electrophoresis (SDS-PAGE). After electrophoresis, the gel was renatured in 2.5% Triton X-100 solution at room temperature for 30 min with gentle agitation, and incubated in developing buffer (50 mmol/L Tris-HCl, pH 7.6; 200 mmol/L NaCl, 5 mmol/L CaCl_2_) at room temperature overnight with gentle agitation. Transparent bands of gelatinolytic activity were visualized by staining with 0.5% Coomassie Blue R250.

### Silver staining and Western blotting

Total proteins (1–5 *μ*g of total proteins; Bradford assay, Biorad Kit, Biorad, Marnes-la-coquette, France) separated on SDS-PAGE, were either silver stained 28 or analyzed using Western blots using the 5ST4A9 or 5ST4C10 6 and 3H3 23 monoclonal antibodies (IGBMC, France). The anti-rab7 rabbit polyclonal antibody (#2576; IGBMC, France) detecting the human ubiquitous Ras-associated RAB7 protein (NM_004637) served as loading control. Quantification was performed using Fiji software 29.

### Mass spectrometry

Protein bands were in-gel reduced, alkylated, and trypsin digested. The extracted peptides were dried (SpeedVac 5301 Concentrator Eppendorf, Hambourg, Germany), resuspended (3 *μ*L 0.1% HCOOH), injected in the nano liquid chromatography, and analyzed on a linear ion trap LTQ XL ETD with nanoESI source interfaced to a nanoLC system (Ultimate 3000; Thermo Fisher Scientific, Germering, Germany). Samples were desalted and concentrated (reverse phase precolumn 300 μm i.d. × 5 μm, 3 min, 20 *μ*L/min), and separated (Pepmap C18, 15 cm × 75 μm i.d). MS/MS spectra were recorded in the data-dependent mode on the five most intense ions observed in the mass range *m*/*z* 350–1600. A micro zoom scan was acquired to determine the peptide charge. Parameters for acquiring collision induced dissociation MS/MS spectra: activation time = 30 msec, activation *Q* = 0.25, relative collision energy = 35%, and an isolation width of 3 *m*/*z*. ETD MS/MS spectra were acquired using: supplemental activation enabled, reaction time = 100 msec, reagent automatic gain control target = 1 E5 ion counts, full AGC target = 3 E4, isolation width of 3 *m*/*z*.

Proteome Discoverer 1.3 (Thermo Fisher Scientific) with Sequest® search engine was used for spectra preprocessing protein ID using the Mouse or the Human Swissprot database (version 15.7). Sequest® (San José, CA) results were filtered with Xcorr versus charge state 1.5–1, 2.5–2, 3–3, 3.2–4.

### HFL1 culture medium and HT-1080 whole cell lysate

Human fetal lung fibroblast HFL1 and fibrosarcoma HT-1080 cell lines (American Type Culture Collection) were cultured in Dulbecco's Modified Eagle's Medium (DMEM; Gibco, Cergy-Pontoise, France), 10% fetal calf serum (FCS) and gentamicin. To improve the production of endogenous MMP-11, HFL1 cells were treated with 12-O-tetradecanoyl phorbol-13-acetate (TPA) (10 ng/mL, 48 h, 0% FCS) 16. HFL1 conditioned media (CM) were centrifuged (5 min, 100 *g*, 4°C), 100-fold concentrated (80% ammonium sulfate) and dialyzed. For whole cell lysates (CL), HT-1080 cells were incubated on ice for 10 min in lysis buffer (50 mmol/L Tris-HCl pH 7.5, 150 mmol/L NaCl, 1 mmol/L ethylenediaminetetraacetic acid, 1% Triton X-100, 1× protease inhibitor cocktail) and centrifuged (10,500 *g*, 10 min, 4°C). Supernatants were recovered.

### HT-1080 knocked-down for MMP-14 expression

MMP-14 was silenced using MMP-14-specific shRNAs (21 nucleotides; RNAi Central Tool; http:hannonlab.cshl.edu/GH_research.htlm) scattered along MMP-14. Oligonucleotides were cloned in XhoI/EcoRI-digested pLMP retroviral vector 30 (pLMP shMMP-14); control vector: 5′-CCAGTCGCCATTATAATGCAA-3′ scramble sequence (pLMP shScr). Positive clones were sequenced. For each shRNA, after retroviral infection (10 *μ*g/mL polybrene, 20 mmol/L HEPES), stably modified cells were puromycin selected and pooled.

### HT-1080 overexpressing MMP-14

Full-length human MMP-14 cDNA was inserted in EcoRI-digested pQCXIP vector (Clontech, Saint-Quentin, France). The empty pQCXIP vector served as control. Retroviruses were generated and, after retroviral infection (10 *μ*g/mL polybrene, 20 mmol/L HEPES), puromycin-selected cells were pooled.

### HFL1 and HT-1080 coculture

Cells cocultured 24 h (1:1 ratio, DMEM, 10% FCS) were TPA treated (10 ng/mL, 48 h, 0% FCS). HFL1 and HT-1080 cells cultured alone served as controls.

### Immunofluorescence analysis

HT-1080 and HFL1 cells were grown on glass coverslips, fixed (10 min, room temperature (RT), 4% paraformaldehyde) and permeabilized (10 min, 0.1% Triton X-100). For MMP-14 cell membrane staining, HT-1080 cells were acid washed (5 min on ice, 50 mmol/L glycine-HCl, pH 3.0, 150 mmol/L NaCl). After several washes (ice-cold, pH 7.4, 0.1 mmol/L CaCl_2,_ 1 mmol/L MgCl_2_), cells were blocked (1% bovine serum albumin) and incubated at RT with 5ST4A9 anti-MMP-11 or MAB3328 anti-MMP-14 (Millipore, Guyancourt, France) mouse monoclonal antibodies, and then with Alexa488-conjugated secondary antibody (Invitrogen-Molecular Probes, Saint-Aubin, France). Nuclei were counterstained with Hoechst-33258 dye. Mounted slides (ProLong Gold; Invitrogen) were observed with a confocal microscope (Leica SP2 UV, Wetzlar, Germany, 63x, NA 1.4).

### Edman analyses

The MMP-11 F1, F2, and F3 fragments (58 pmol MMP-11 digested using 58 pmol MMP-14, 15 min, RT) were separated by SDS-PAGE (F1 and F2: 10%; F3: 15-20% gradient), and transferred on polyvinylidine difluoride (PVDF) membranes. The Coomassie Blue-stained PVDF bands were excised and submitted to N-terminal sequence analyses according to Edman chemistry (ABI Procise 492HT peptide sequencer; Applied Biosystems, Foster City, CA).

### Results and statistical analyses

For each study, at least three independent experiments were performed. All of them gave similar results. Moreover, MMP-11 and MMP-14 enzymatic activity analyses were performed in triplicate and statistical differences were evaluated using student's *t* test. *P* values lower than 0.05 were considered as significant. *P < 0.05; **P < 0.01; ***P < 0.001.

## Results

### MMP-11 and MMP-14 exhibit similar spatiotemporal expression at the tumor invasive front

**Figure 1:**
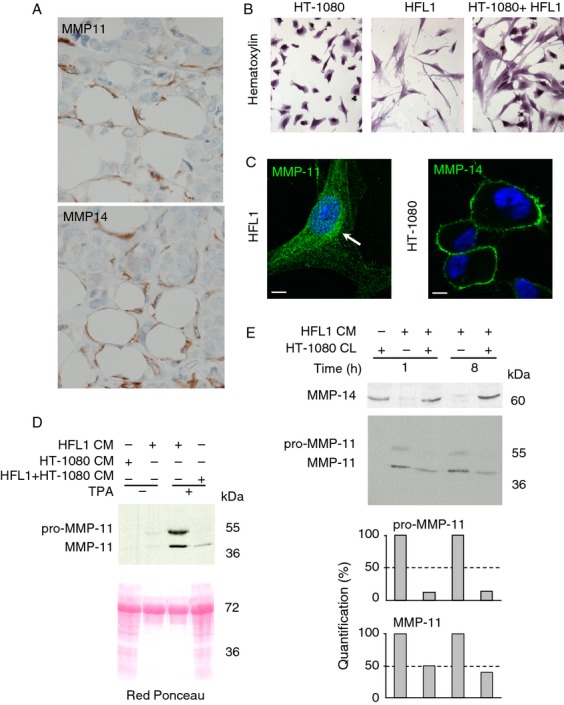
In vivo and in vitro MMP-11 and MMP-14 expression. (A) MMP-11 and MMP-14 immunohistochemistry of serial sections from an human invasive front of breast carcinoma using 5ST4A9 and 3H3 antibodies, respectively. Both MMPs (brown) were present in fibroblasts and CAAs (lipids in white) located adjacent to invading cancer cells showing that, in vivo, MMP-11 and MMP-14 are close proteins. Nuclei were in blue. Original magnification: 400×. (B) Hematoxylin staining of HT-1080 and HFL1 cells grown either alone or together. (C) Immunofluorescence analyses visualized MMP-14 at the HT-1080 cell membrane (green) and MMP-11 in the HFL1 secretory pathway (green, white arrow). Nuclei are in blue. Bars: 2 microns. (D) Coculture of the two cell lines led to a dramatic decreased levels of MMP-11 in the CM. Red Ponceau stained the secreted proteins. (E) Western blot of HFL1 CM (10 *μ*g protein total containing HFL1-produced MMP-11) and HT-1080 CL (90 *μ*g protein total containing HT-1080-produced MMP-14) incubation mixtures (1 and 8 h, RT). More than 50% of the pro-MMP-11 and 50% of MMP-11 were hydrolyzed after 1 h. MMP-14 remained similar in the presence or in the absence of HFL1 CM and whatever the MMP-11 levels, indicating that MMP-11 does not cleave MMP-14. Data from one representative experiment. CAA, cancer-associated adipocytes; CM, conditioned media; CL, cell lysates; MMP-14, matrix metalloproteinase-14.

We hypothesized that a functional relationship might exist between MMP-11 and MMP-14. This could be very important in malignant conditions since MMP-11 was shown to play an essential role during the early steps of cancer cell invasion of adjacent connective tissues 1,5,6. MMP-14 has been reported to be expressed by both stromal and cancer cells 22,23. Thus, we checked for the presence of MMP-14 in the MMP-11-expressing area. MMP-11 and MMP-14 immunohistochemistry was performed on serial sections of human breast cancer biopsies. As expected from previous data 6, peritumoral fibroblasts and cancer-associated adipocytes (CAAs) that are adipocytes located at the cancer cell contact 31, expressed MMP-11 at the tumor invasive front. Interestingly, MMP-14 staining gave similar images (Fig. 1A). This was observed in all samples examined (more than 20) indicating that this is a common event during local invasion.

Thus, at least during early desmoplasia, MMP-11 and MMP-14 are physically very close supporting a possible MMP-11/MMP-14 interaction.

### HT-1080 and HFL1 cell coculture leads to MMP-11 decrease

Since MMP-11/MMP-14 interaction is quite impossible to study in vivo, we presumed that similar dynamic events should occur in vitro. We used HT-1080 fibrosarcoma and HFL1 nontumoral stromal cell lines grown in serum-free conditions (Fig. 1B). As expected, immunofluorescence experiments showed MMP-14 at the plasma membrane of HT-1080 cells and MMP-11 in the secretory pathway of HFL1 cells (Fig. 1C). HFL1 cells were boosted for MMP-11 expression by TPA treatment 16 (Fig. 1D). Western blot analyses of HFL1 cell culture medium (CM; containing secreted MMP-11) using the 5ST4A9 antibody directed against human MMP-11 showed two bands (55 kDa and 47 kDa) corresponding to the pro- and mature forms of MMP-11, respectively 16. Interestingly, coculture of the two cell lines led to a dramatic decrease in MMP-11 compared with the levels observed when HFL1 cells were grown alone, indicating the occurrence of MMP-11 proteolysis (Fig. 1D). Then, HT-1080 whole cell lysate (CL; containing membrane-bound MMP-14) and HFL1 CM from cells grown alone were incubated together for varying times. Western blot analysis with the 3H3 antibody recognizing MMP-14 showed no decrease in MMP-14, indicating that HFL1 CM did not contain enzyme(s) affecting MMP-14. However, more than 50% decrease in pro-MMP-11 and MMP-11 was seen in the presence of HT-1080 CL. This effect was already complete after 1 h of incubation (Fig. 1E).

Thus, HT-1080 CL contains enzyme(s) able to efficiently cleave MMP-11.

### HT-1080-produced MMP-14 cleaves recombinant FL-MMP-11

**Figure 2:**
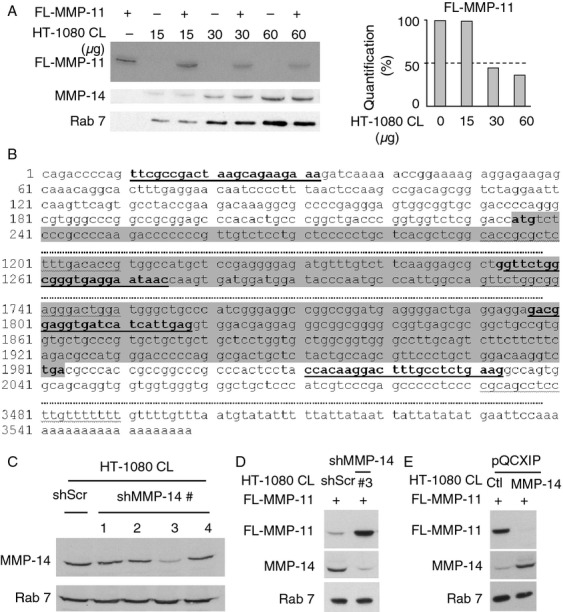
Analysis of cleavage of recombinant FL-MMP-11 by HT-1080-produced MMP-14. (A) Western blot of FL-MMP-11 (90 nmol/L) incubated with HT-1080 CL (15–60 *μ*g protein total) showed a dose-dependent decrease in FL-MMP-11 intensity, indicating that HT-1080 CL has enzymatic activity toward MMP-11. No MMP-11 was present in HT-1080 CL. MMP-14 levels remained constant whatever the FL-MMP-11 status, indicating that MMP-11 does not cleave MMP-14. (B) The four putative MMP-14 shRNA target sequences of human MMP-14 mRNA (underlined, #1 to #4 5′–3′); ATG and TGA are in bold, coding sequence in grey. (C) CL Western blot showed that MMP-14 was dramatically reduced in HT-1080/shMMP-14#3 cell pool. (D) FL-MMP-11 (90 nmol/L) incubation with HT-1080/shScr CL (90 *μ*g protein total; 2 h; RT) led to its decrease but not with HT-1080/shMMP-14#3 CL. (E) FL-MMP-11 (90 nmol/L) incubation with HT-1080/pQCXIP-MMP-14 CL (90 *μ*g protein total; 2 h; RT) decreased FL-MMP-11 level compared with HT-1080/pQCXIP CL. Thus, FL-MMP-11 cleavage is MMP-14 dependent. Rab 7 serves as loading control. (A and C–E) Data from one representative experiment. CL, cell lysates. MMP-14, matrix metalloproteinase-14.

To check if HT-1080-produced MMP-14 is involved in MMP-11 proteolysis we produced human Full-length MMP-11 (FL-MMP-11) recombinant protein (active form; 47 kDa) as reported 24–27. A constant quantity of FL-MMP-11 was incubated with increasing quantities of HT-1080 CL. Western blot analysis showed that the band intensity of FL-MMP-11 decreased with increasing quantities of HT-1080 CL, confirming that HT-1080 CL contained enzyme(s) able to hydrolyze MMP-11 (Fig. 2A). To study MMP-14 loss-of-function, HT-1080 cells were modified using pLMP retroviral vectors expressing four MMP-14-specific shRNA sequences (Fig. 2B), or scramble shRNA (shScr; negative control). A pool of MMP-14-silenced HT-1080 cells was established for each shMMP-14 (HT-1080/shMMP-14#1 to #4) and for shRNAScr (HT-1080/shScr). CL from the 5 pools were analyzed by western blot (Fig. 2C). HT-1080/shScr expressed normal MMP-14 levels. HT-1080/shMMP-14#3 pool of cells that most efficiently reduced MMP-14 expression was chosen for further experiments. Interestingly, FL-MMP-11 was decreased after incubation with HT-1080/shScr CL but not with HT-1080/shMMP-14#3 CL (Fig. 2D). Finally, to perform gain-of-function experiments, we developed HT-1080 cells overexpressing MMP-14 using pQCXIP retroviral vector. As expected, HT-1080/pQCXIP-MMP-14 CL induced an increased MMP-11 proteolysis compared with control HT-1080/pQCXIP CL containing the vector alone (Fig. 2E).

Altogether, these data identify MMP-11 as a substrate of MMP-14.

### Recombinant Cat-MMP-14 cleaves HFL1-produced MMP-11

**Figure 3:**
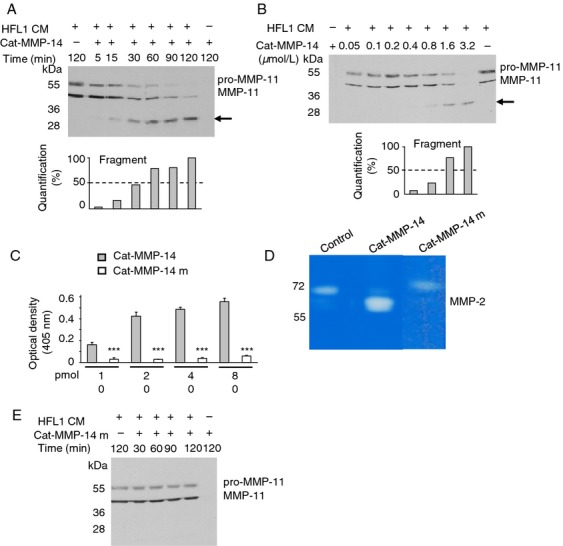
Analysis of cleavage of HFL1-produced MMP-11 by recombinant Cat-MMP-14. (A) HFL1 CM (10 *μ*g protein total) were incubated with buffer alone or with Cat-MMP-14 (6.5 *μ*mol/L) for increasing times (5–120 min; RT). Mixtures were electrophoresed (10% SDS-PAGE). Western blotting visualized the pro-MMP-11 and MMP-11 bands. One additional lower band appeared after 5 min and rose 50% of intensity around 45 min incubation (black arrow). Concomitantly, the 2 MMP-11 bands progressively disappeared. (B) Increasing amounts of Cat-MMP-14 (0.05–3.2 *μ*mol/L) incubated with constant HFL1 CM quantity (10 *μ*g protein total; 2 h; RT) showed lower band appearance and increase from 0.4 to 3.2 *μ*mol/L of Cat-MMP-14 (black arrow), whereas pro-MMP-11 and MMP-11 disappeared. (C) Mutated Cat-MMP-14 m had no enzymatic activity toward a1-PI compared with the active Cat-MMP-14 (*P* < 0.001). (D) cat-MMP-14 (0.5 *μ*g) and cat-MMP-14 m (0.5 *μ*g) activity were assayed by zymography. U87MG-produced pro-MMP-2 was activated by cat-MMP-14, whereas cat-MMP-14 m had no effect compared with the control (no MMP-14 incubation). (E) In experimental conditions similar to (A), Cat-MMP-14 m had no effect. (A–E) Data from one representative experiment. HFL1, human fetal lung fibroblast. CM, conditioned media.

To further study the MMP-14 cleavage, we produced the human MMP-14 recombinant catalytic domain (Cat-MMP-14), and test its activity against HFL1-produced MMP-11. A constant quantity of HFL1 CM was first treated with vehicle alone or with a constant Cat-MMP-14 quantity for various times. Western blotting analysis of the resulting mixtures showed that the pro-MMP-11 and MMP-11 bands progressively disappeared with increasing incubation times. The pro-form was completely cleaved after 120 min of reaction. Concomittantly a lower band recognized by the 5ST4A9 antibody appeared at 32 kDa whose intensity increased and became optimal after 120 min. Half of its production was shown after 45 min (Fig. 3A). Then, HFL1 CM was incubated with increasing quantities of Cat-MMP-14. The two MMP-11 forms decreased when Cat-MMP-14 quantities increased. Incubation with 3.2 *μ*mol/L of Cat-MMP-14 led to the complete disappearance of the pro-MMP-11 and MMP-11. Moreover, half of the intensity of the 32 kDa MMP-11 fragment was obtained by about 1.2 microM MMP-14 (Fig. 3B). Finally, to determine if MMP-11 cleavage was directly due to MMP-14 enzymatic activity, a point-mutated form of Cat-MMP-14 (Cat-MMP-14 m) was designed and produced. Cat-MMP-14 m was devoid of enzymatic activity using both a1-PI (Fig. 3C) and pro-MMP-2 activation (Fig. 3D) tests. No MMP-11 cleavage was obtained using Cat-MMP-14 m (Fig. 3E), indicating that Cat-MMP-14 cleaves HFL1-produced MMP-11 via its catalytic function.

These results indicated that MMP-14 cleaves MMP-11 in a time- and dose-dependent manner.

### MMP-11 contains two cleavage sites for MMP-14

**Figure 4:**
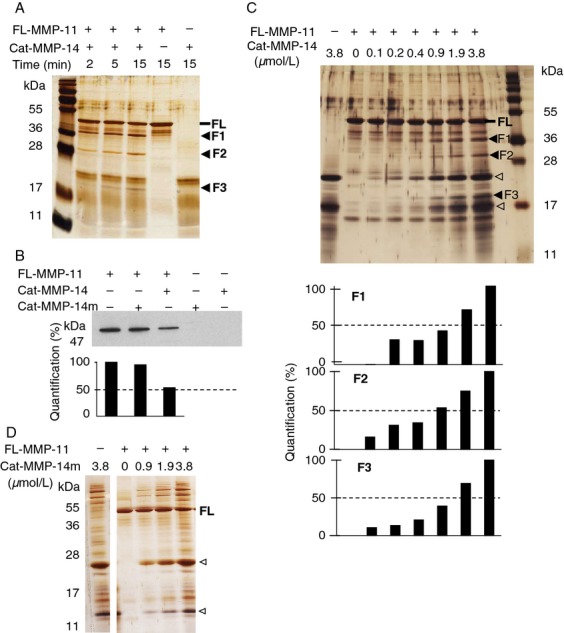
Analysis of recombinant FL-MMP-11 cleavage by recombinant Cat-MMP-14. (A) FL-MMP-11 (FL; 1.9 *μ*mol/L) and Cat-MMP-14 (1.9 *μ*mol/L) proteins were analyzed separately, and after stoichiometric incubation (RT, various times, 12% SDS-PAGE). Silver nitrate staining showed appearance of three main bands after coincubation (F1–F3, black arrowheads). (B) Western blot showed 50% disappearance of FL-MMP-11 after its stoichiometric incubation with Cat-MMP-14 (1.9 *μ*mol/L per MMP, 1 h, RT). Inactive Cat-MMP-14 m had no effect. (C) Kinetic experiment incubating FL-MMP-11 (1.9 *μ*mol/L) with increasing amounts of Cat-MMP-14 (empty arrowheads; 0.1–3.8 *μ*mol/L, 15 min, RT; 12.5–20% gradient SDS-PAGE; silver nitrate). Quantification showed that F1–F3 production increased depending on Cat-MMP-14 quantities. Bands start to appear for 0.1 *μ*mol/L of Cat-MMP-14, and 50% intensity was risen for 0.9–1.9 *μ*mol/L depending on the band observed. (D) In similar experimental conditions (15–12.5% gradient gel), inactive Cat-MMP-14 m had no effect. (A–D) Data from one representative experiment. MMP-14, matrix metalloproteinase-14.

The cleavage sites were investigated using recombinant proteins. Silver nitrate staining showed one main band at 47 kDa for FL-MMP-11, and at 22 kDa for Cat-MMP-14, as reported 24–27. FL-MMP-11 incubation with Cat-MMP-14 in a stoichiometric manner for increasing times led to three main bands numbered F1–F3 at 36, 29, and 19 kDa, respectively (Fig. 4A). They already appeared at 2 min incubation and their intensity increased with incubation time. F1 and F2 bands were obviously MMP-11-related (sizes higher than Cat-MMP-14). F3 analysis by nano liquid chromatography—mass spectrometry using a LTQ XL ETD mass spectrometer (Thermo Fisher Scientific, San José, CA) showed that it corresponded to MMP-11 fragment (data not shown) confirming that MMP-14 cleaves MMP-11 but the reverse is not true. Consistently, western blot analysis of sample mixtures showed a decrease in the FL-MMP-11 band (Fig. 4B). Then, a constant quantity of FL-MMP-11 was incubated with varying Cat-MMP-14 quantities. F1–F3 firstly appeared at a 20:1 FL-MMP-11:Cat-MMP-14 substrate:enzyme relative molar ratio. Setting the band intensity obtained for the highest Cat-MMP-14 concentration at 100%, half of the F1–F3 bands were produced for 1:2 to 1:1 MMP-11:MMP-14 molar ratio (Fig. 4C). Cat-MMP-14 m had no effect (Fig. 4B and D).

Thus, MMP-11 cleavage by MMP-14 leads to three major protein fragments indicating the presence of two cleavage sites.

### MMP-14 cleaves MMP-11 at its catalytic domain

**Figure 5:**
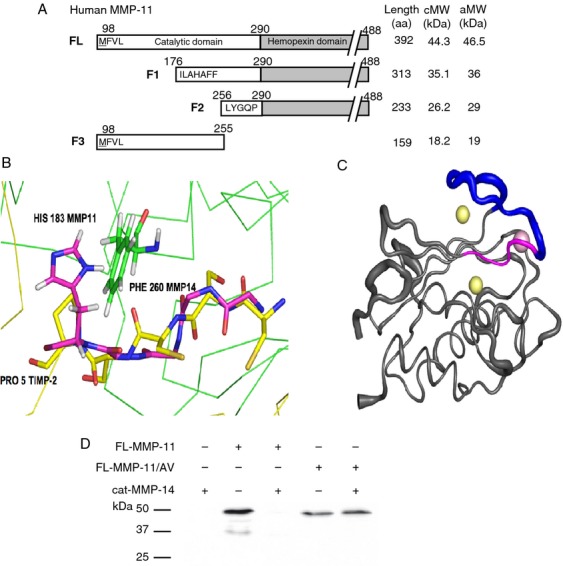
Identification of cleavage sites and molecular modeling of MMP-11/MMP-14 interaction. (A) F1 to F3 N-ter amino acid sequences established using Edman analysis. aa, number of residues; cMW and aMW, calculated and apparent fragment molecular weight (coomassie blue or silver nitrate), respectively. (B) 3D structures of MMP-11 (PDB ID: 1HV5) 33 and MMP-14 (PDB ID: 1BQQ) 32. All calculations were done using the CHARMM program with the all-atom 27 protein force field 47. Initial hydrogen positions were calculated using the HBUILD facility 48. Synthetic and protein ligands were removed, as well as crystallographic water molecules. Cartoon showing the PGG(P1)-I(P1′)LA cleavage site (situated in N-ter of strand IV 33) inside the MMP-14 active site compared with the TIMP2/MMP-14 complex. TIMP-2 is represented as a yellow Calpha trace, excepted for the sequence CSCSP (all atoms shown) and MMP-14 as a green Calpha trace, excepted the Phe260 (all atoms shown). TIMP-2 residues CSCSP placed in the active site of MMP-14 superpose to MMP-11 residues GILAH (179–183); only the backbone atoms are shown (carbon in pink), excepted the His183 (side chain shown). MMP-11 His 183 (superposed to TIMP-2 Pro 5) is naturally close to Phe 260 of MMP-14 with which it could form aromatic stacking. (C) Root Mean Square fluctuations of MMP-11, obtained from normal mode analysis using the VIBRAN module of the CHARMM program 49 are depicted on the MMP-11 backbone. Increased width of the cartoon representation indicates regions of higher flexibility, in particular, the long S-shaped loop, situated N-ter to the cleavable site, even while coordinating the structural zinc and calcium ions. MMP-11 GILAH (179–183) in pink; S-shaped loop (165–178) in deep blue; zinc and calcium ions, yellow and pink spheres, respectively. (D) Cat-MMP-14 does not cleave recombinant FL-MMP11/AV (GI to AV mutant). Western blot analysis using 5ST4C10 monoclonal antibody recognizing MMP-11 showed that FL-MMP-11/AV was no more digested by Cat-MMP-14. Data from one representative experiment. MMP-14, matrix metalloproteinase-14.

Cleavage site motif for a MMP involves residues both N- and C-terminal to the scissile bond. Human F1–F3 fragments were subjected to Edman sequencing analysis to identify their N-terminus sequences (N-ter). The N-ter of F1, F2, and F3 was ILALAFFRL, LXGQPXPTVT, and MFVLSG, respectively (Fig. 5A). These data allowed to map the two MMP-14 cleavage sites along the MMP-11 sequence, between glycine 175 and isoleucine 176, and between histidine 255 and leucine 256. Interestingly, both PGG(P1)-I(P1′)LA and VQH(P1)-L(P1′)YG sites are located in the catalytic domain of MMP-11. Similar mouse MMP-11 F1–F3 fragments were generated by Cat-MMP-14 cleavage (data not shown). Sequence analysis and Edman determination showed that MMP-14 cleaved the mouse MMP-11 between glycine 179 and isoleucine 180, and between histidine 259 and leucine 260 revealing similar PGG(P1)-I(P1′)LA and IQH(P1)-L(P1′)YG cleavage sites in the mouse MMP-11 catalytic domain.

Virtually all MMPs form tight 1:1 complexes with the TIMPs. The structure of the catalytic domain of MMP-14 in complex with TIMP-2, a natural MMP-14 inhibitor, is known 32. We used it to guide a rigid body positioning of MMP-11 33 inside the MMP-14 active site. Interestingly, molecular modeling showed the superposition of residues of the cleavable MMP-11 PGG-ILA sequence and TIMP-2 (Fig. 5B). Thus, TIMP-2 residues CSCSP placed in the active site of MMP-14 are shown to superpose to MMP-11 residues GILAH (179–183). It also indicated that conformational flexibility of MMP-11, particularly on the N-terminal side of the cleavage site, is a prerequisite to the cleavage of MMP-11 by MMP-14. Conformational flexibility of MMP's has been documented 34,35. Thus, an nuclear magnetic resonance study of human stromelysin-1 34 indicated that in the absence of inhibitor, the protein is highly flexible. Interestingly, the structure of the catalytic domain of MMP-11 indicates that this cleavable site is situated in the *β* strand IV, immediately C-terminal to the S-shaped loop 33 and the normal mode calculations indicate that this region displays important conformational flexibility (Fig. 5C).

These structural insights prompted us to further investigate the importance of the G and I residues located in P1 and P1′ of the PGG-ILA cleavage site for cleavage efficiency. We produced recombinant FL-MMP-11 mutant where we replaced GI by AV (FL-MMP-11/AV) and tested the activity of Cat-MMP-14 on this mutant. Interestingly, western blot analysis showed that FL-MMP-11/AV cleavage was dramatically reduced compared to FL-MMP-11 (Fig. 5D). Together, these results showed that the GI sequence is essential for MMP-11 cleavage by MMP-14.

Thus, MMP-14 cleaves MMP-11 within its catalytic domain, and this function is conserved between human and mouse.

### MMP-14 inactivates MMP-11 enzymatic activity

**Figure 6:**
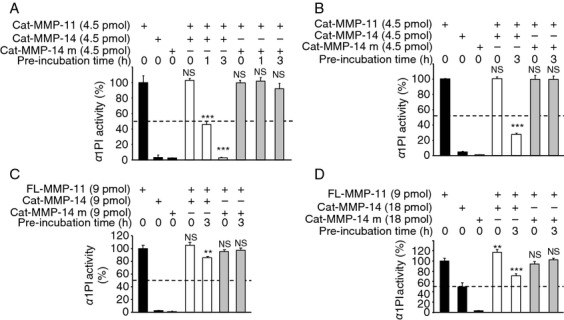
Analysis of MMP-11 enzymatic activity toward a1-PI after MMP-14 cleavage. (A) Human Cat-MMP-11: We used 4.5 pmoles of each MMP, since at this concentration Cat-MMP-11 was highly active compared with Cat-MMP-14. No Cat-MMP-11 inactivation was observed when both MMPs were added together without preincubation (0 h). However, 1 h preincubation led to about 50% decrease of Cat-MMP-11 activity (*P* < 0.001), and after 3 h Cat-MMP-11 was no longer active (*P* < 0.001). (B) Mouse Cat-MMP-11: Cat-MMP-14 dramatically reduces its activity, about 70% after 3 h of preincubation (*P* < 0.001). (C and D) Mouse FL-MMP-11. (C) We used 9 pmoles of each MMP, since at this concentration FL-MMP-11 was highly active compared with Cat-MMP-14. In the absence of preincubation, FL-MMP-11 activity remained similar. By contrast, after preincubation, lower enzymatic activities was observed. Cat-MMP-14 decreases FL-MMP-11 function by 20% after 3 h of preincubation. (D) We then used 9 pmoles of FL-MMP-11 and 18 pmoles of Cat-MMP-14. At this concentration, Cat-MMP-14 alone exhibited half of the FL-MMP-11 activity toward a1-PI. In the absence of preincubation, cumulative enzymatic activity of FL-MMP-11 and Cat-MMP-14 was observed indicating that the two enzymes remained active. By contrast, after preincubation, Cat-MMP-14 decreases FL-MMP-11 function by at least 40% after 3 h of preincubation. Black bars: basal activities of MMPs alone; white bars: MMP-11 incubated with active Cat-MMP-14; grey bars: MMP-11 incubated with inactive Cat-MMP-14 m. In all cases, Cat-MMP-14 m had no effect. NS, not significant. (A–D) Data from one representative experiment. MMP-14, matrix metalloproteinase-14.

We first checked if cleavage by MMP-14 affects human MMP-11 enzymatic function using the a1-PI quantitative colorimetric assay. The Cat-MMP-11 and Cat-MMP-14 catalytic domains were used alone or after preincubation (Fig. 6A). Cat-MMP-11 alone was greatly more efficient to cleave a1-PI than Cat-MMP-14 when tested alone as previously reported 24. When preincubated together, Cat-MMP-11 activity was dramatically reduced. More than 50% was decreased after 1 h of preincubation (*P* < 0.001), and it was completely abolished after 3 h (*P* < 0.001). On the contrary, when Cat-MMP-11 and Cat-MMP-14 were tested together but without preincubation (0 h), the level of a1-PI cleavage remained constant indicating that Cat-MMP-11 was still active (Fig. 6A). Inactive Cat-MMP-14 m had no effect. The human MMP-11 presents a characteristic that is the replacement of a canonical Proline, found in almost all catalytic domain of MMPs including mouse MMP-11, by an Alanine (Ala-235) 1,33. To test the impact of this difference, similar experiments were conducted with mouse Cat-MMP-11. Similar results were obtained (Fig. 6B) indicating that this residue is not important for MMP-14 site recognition/function.

Finally, to study the impact of the hemopexin domain, we tested the MMP-14 activity on the full-length form of MMP-11 using the mouse FL-MMP-11 (Fig. 6C and D). Using same quantity of FL-MMP-11 and Cat-MMP-14, when added without preincubation, mixture was as efficient as FL-MMP-11 alone. However, Cat-MMP-14 preincubation led to a significant 20% decrease in FL-MMP-11 activity (*P* < 0.01). We then used twofold more Cat-MMP-14 than FL-MMP-11. At this concentration, Cat-MMP-14 activity alone was about half of that of FL-MMP-11 when tested alone (Fig. 6D). When added without preincubation, mixture efficiency was increased in comparison with FL-MMP-11 alone, indicating that both MMPs were still active, and their enzyme activities were cumulated (*P* < 0.01). By contrast, after preincubation, the FL-MMP-11 inactivation was more pronounced (40%; *P* < 0.001). It should be noticed that this value is greatly underestimated since it includes the intrinsic Cat-MMP-14 activity. Cat-MMP-14 m had no activity. Thus, MMP-14 also inactivates the full-length MMP-11 activity, indicating that the hemopexin domain does not greatly altered MMP-14 site recognition/function.

Collectively, these data indicate that MMP-14 inactivates enzymatic activity of the full-length or C-terminally truncated MMP-11, regardless their human or mouse origin.

## Discussion

In this study, we demonstrate that MMP-11 is a substrate of MMP-14. MMP-14 cleaves human and mouse MMP-11 between the G and I residues of the PGG-ILA, and the H and L of V/IQH-LYG conserved sequences, both located in the catalytic domain. This cleavage leads to the MMP-11 inactivation. Since MMP-14 is produced by HT-1080 cancer cells, whereas MMP-11 is secreted by HFL1 stromal cells, our findings support the emerging importance of tumor-stroma interaction/cross-talk. These data are of interest since MMP-11 and MMP-14 are potent factors of the pericellular microenvironment in a broad spectrum of biological events, including cancer cell invasive steps.

That MMP-14 cleaves other MMPs is not new. Many reports have shown that MMP-14 processes several pro-MMPs leading to their activation: pro-MMP-8 36, pro-MMP-13 37, and most notably pro-MMP-2 18,21,38,39. MMP-14 is therefore involved in multiple zymogen activation pathways. By contrast, we now show that MMP-14 hydrolyzes an active MMP and negatively regulates its enzymatic activity through direct cleavage of the catalytic domain. Accordingly, autocatalytic MMP-14 cleavage on the cell surface leading to an inactive membrane-tethered species has been reported 40–42. Thus, MMP-14-dependent hydrolysis of MMP might be another way to control the extent of one MMP activity, independently of the canonical reversible TIMP-dependent inhibitory process. Interestingly, it was reported recently that MT1-MMP proteolytically inactivates a disintegrin and metalloproteinase 9 (ADAM9), a member of another type of metalloproteinases 43.

In addition to pro-MMPs, other substrates including the interstitial collagen, adhesive glycoproteins and proteoglycans, latent enzymes and growth factors, chemokines, and more recently intracellular substrates have been reported for MMP-14 12. A survey of known cleavage sites showed that MMP-14 recognizes various sequences. PxG/P(P1)-L(P′1) and PL(G/P/A)(P1)-L(P1′)(R/M) consensus sequences were defined using bacteriophage peptide display libraries 44. Although original, the PGG(P1)-I(P′1)LA and V/IQH(P1)-L(P1′)YG cleavage sites are partially conserved, like the P and G residues in P3 and P1 for the first site, and P and L residues in P3 and P1′ for the second. A search of the Blast protein sequence database shows that PGG-ILA sequence is only present in MMP-11, whereas V/IQH-LYG is shared with MMP-26. Whether MMP-14 cleaves MMP-26 remains to be studied.

The crystallographic structure of the mouse MMP-11 catalytic domain complexed to RXPO3, a phosphinic inhibitor mimicking the transition-state, shows MMP-11 to have five-stranded beta-sheets and three alpha helices 33. It is a negatively charged globular protein containing several pockets. The S1′ subsite, a well defined hydrophobic pocket of variable depth depending on the MMP, is crucial for the specificity of the enzymatic activity of MMPs. The S1′ MMP-11 pocket is larger than those of the other MMPs due to the presence of an E at position 215 33. Interestingly, we observed that the G(P1) identified in this study is one of the residues involved in Ca^++^ binding and which contributes to the correct structure of the S1′ MMP-11 pocket. This suggests that part of the catalytic site of MMP-11 serves as cleavage site for MMP-14.

MMP-11 cleavage by MMP-14 generates a 36 kDa MMP-11 fragment consistent with the size expected for the MMP-11 F1 fragment from the PGG-ILA cleavage. Interestingly, Mari et al. 19 have previously reported, in a tumor/stroma cell coculture assay, that the released active form of MMP-11 is processed via an unidentified MMP-dependent mechanism to a 35 kDa protein lacking enzymatic activity. The authors identified several sites of cleavage located C-terminal to the PGG-ILA sequence. It is therefore tempting to speculate that the 35 kDa MMP-11 form reported by these authors might result from PGG-ILA MMP-14-mediated cleavage.

MMP-14 is a transmembrane protein while MMP-11 is soluble into the cellular microenvironment. By hydrolyzing MMP-11, membranous MMP-14 might provide spatial restriction of MMP-11 proteinase activity and locally protect specific and vital pericellular ECM and cells from the inappropriate function of MMP-11.

That MMP-14 negatively regulates MMP-11 activity is consistent with the spatial expression pattern that both proteins share. In addition, a number of in vivo functional observations show that MMP-14 acts often in an opposing manner to MMP-11. For example, it has been shown that MMP-14 coordinates adipocyte differentiation by acting as an inducer of adipogenesis 45, whereas MMP-11 induces adipocyte dedifferentiation and acts as a negative regulator of adipogenesis 6,46. Therefore, one of the main questions is to identify which MMP-14 function depends on MMP-11 inactivation. While pro-MMP activation or collagen cleavage by MMP-14 seems to be MMP-11-independent, this is less evident during adipogenesis, where MMP-14 might balance MMP-11 function. Moreover, since MMP-14 has numerous substrates, dynamic competitive processes might exist between them and MMP-11. The relative level of in vivo interaction between these two proteins remains to be determined.

In conclusion, the present work brings new insight into the critical regulatory mechanisms of MMP-11 function. Our data also highlight the continuously increasing complexity of the MMP network. To date, MMP-14 was known as an activator of MMP function. Our results indicate that MMP-14 is also a negative regulator of MMP function. This further reinforced the concept of MMP-14 as a central player in the MMP cascade.
